# Unveiling Fermentation Effects on the Functional Composition of Taiwanese Native Teas

**DOI:** 10.3390/molecules31010171

**Published:** 2026-01-01

**Authors:** Wei-Ting Hung, Chih-Chun Kuo, Jheng-Jhe Lu, Fu-Sheng Yang, Yu-Ling Cheng, Yi-Jen Sung, Chiao-Sung Chiou, Hsuan-Han Huang, Tsung-Chen Su, Hsien-Tsung Tsai, Kuan-Chen Cheng

**Affiliations:** 1 Institute of Food Science and Technology, National Taiwan University, No. 1, Sec. 4, Roosevelt Rd., Taipei 10617, Taiwan; milahung4123@gmail.com; 2 Tea and Beverage Research Station, Ministry of Agriculture, No. 324, Chung-Hsing Rd., Taoyuan 326011, Taiwan; kcc0204@tbrs.gov.tw (C.-C.K.); hey911s@gmail.com (F.-S.Y.); yulingfish@gmail.com (Y.-L.C.); tres2048@tbrs.gov.tw (Y.-J.S.); chiou-sung@tbrs.gov.tw (C.-S.C.); a1009@tbrs.gov.tw (H.-H.H.); tres201@tbrs.gov.tw (T.-C.S.); 3 Institute of Biotechnology, National Taiwan University, No. 1, Section 4, Roosevelt Rd., Taipei 10617, Taiwan; lusanity40@gmail.com; 4 Department of Optometry, Asia University, No. 500, Lioufeng Rd., Wufeng, Taichung 413305, Taiwan; 5 Department of Medical Research, China Medical University Hospital, China Medical University, No. 91, Hsueh-Shih Road, Taichung 404333, Taiwan; 6 Department of Food Science, Fu Jen Catholic University, No. 510, Zhongzheng Rd., New Taipei City 242062, Taiwan

**Keywords:** Taiwanese native teas, tea processing stages, enzymatic oxidation, catechin oxidation, theasinensins, POPCs

## Abstract

Tea’s chemical composition is influenced by cultivar, harvest maturity, and growing environment; however, processing remains the dominant factor shaping final quality. Despite the diversity of Taiwanese native teas, systematic comparisons of functional components across multiple manufacturing stages remain limited. In this study, nine representative Taiwanese teas were evaluated at four key processing stages—green tea (G), enzymatic fermentation (oxidative fermentation, F), semi-finished tea prior to roasting (S), and completed tea (C)—to clarify how enzymatic oxidation, rolling, and roasting alter major bioactive constituents. Green-tea-stage samples exhibited clear cultivar-dependent profiles: large-leaf cultivars contained higher catechins and gallic acid, whereas bud-rich small-leaf teas showed elevated caffeine and amino acids, with amino acids further enhanced at higher elevations. Fermentation intensity governed the major chemical transitions, including catechin depletion, gallic acid formation, accumulation of early stage catechin-derived paired oxidative polymerization compounds (POPCs), and pronounced increases in theasinensins in heavily fermented teas. L-theanine decreased most markedly in teas subjected to prolonged withering. Roasting further reduced amino acids but had minimal influence on caffeine, while rolling effects varied by tea type. Overall, this study provides the first stage-resolved chemical map of Taiwanese native teas, offering practical insights for optimizing processing strategies to enhance functional phytochemical profiles.

## 1. Introduction

Tea is one of the most widely consumed beverages worldwide and contains a diverse array of bioactive constituents, including catechins, gallic acid (GA), flavonols, amino acids, caffeine, and volatile compounds [1,2]. The chemical profile of tea is shaped by numerous factors such as cultivar, harvest season, agronomic management, climate, soil characteristics, and plucking maturity, while processing methods exert the strongest influence on its final composition [3,4]. Although a broad range of specialty teas is produced in Taiwan, spanning non-fermented, partially fermented, and fully fermented categories [5], comprehensive evaluations of functional components across defined processing stages remain limited. Systematic characterization of representative tea types at key manufacturing steps is therefore essential for clarifying how bioactive compounds are formed, degraded, or transformed during production.

Among the many factors influencing tea chemistry, the manufacturing sequence, which includes withering, rolling, fermentation (enzymatic oxidation), and roasting, produces the most extensive biochemical transformations [6]. During this enzymatic fermentation process, catechins undergo enzymatic oxidation to generate quinone intermediates that couple to produce theaflavins, thearubigins, and other early stage oxidative dimers [7]. A substantial portion of the catechin pool is further transformed into a broad spectrum of secondary polyphenols, which contributes to the sensory and functional characteristics of fermented teas [8]. After fermentation, prolonged thermal treatment during roasting can modify flavonoids, accelerate amino acid degradation, and promote the release of GA from gallated catechins [9]. Collectively, these transformations highlight the central role of processing conditions in shaping the chemical composition and functional properties of finished teas.

Although these mechanisms have been examined individually within specific tea categories, a comprehensive, stage-resolved chemical analysis across multiple Taiwanese native teas remains insufficiently explored. Taiwan produces a uniquely diverse set of teas, including non-fermented green teas, lightly oxidized oolong teas, and highly fermented Oriental Beauty and black teas [10,11,12,13,14]. However, limited research to date has systematically compared their chemical transitions across key processing stages or examined the formation of paired oxidative polymerization compounds (POPCs) and theasinensins under real manufacturing conditions. In this study, the term POPCs is used as an operational descriptor to denote a group of early stage catechin-derived oxidative coupling products formed during tea fermentation through enzymatic oxidation and quinone-mediated reactions. These compounds represent low-molecular-weight oxidative dimers and related coupling products that arise along established catechin oxidation pathways, prior to or concomitantly with the formation of more extensively characterized tea pigments, particularly theasinensins. Under stronger oxidation conditions, such oxidative intermediates may further contribute to the formation of downstream pigments such as theaflavins, as reported in previous studies [8,15,16].

**Figure 1:**
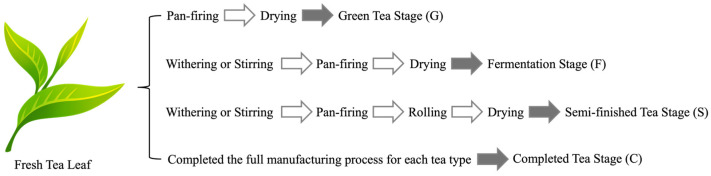
Schematic overview of tea manufacturing processes and the corresponding sampling points (G, green tea stage; F, fermentation stage; S, semi-finished tea prior to roasting; C, completed tea) applied to the Taiwanese native teas evaluated in this study.

Accordingly, this study investigated the chemical transformations occurring throughout the manufacturing process of nine representative Taiwanese native teas, using staged sampling at four key points: green tea (G), fermentation (F), semi-finished tea prior to roasting (S), and completed tea (C). By quantifying catechins, GA, caffeine, free amino acids, POPCs, and theasinensins across key processing stages, this study establishes the first comprehensive process-resolved chemical dataset for Taiwanese teas, providing mechanistic insight and a foundation for refining future manufacturing practices. A schematic overview of the tea manufacturing workflow and the corresponding sampling points (G, F, S, and C) is provided in Figure 1. Accordingly, the objectives of this study were to: (i) systematically characterize the stage-resolved changes in major bioactive constituents—including catechins, gallic acid, free amino acids, caffeine, paired oxidative polymerization compounds (POPCs), and theasinensins—across four key processing stages of Taiwanese native teas; (ii) elucidate how enzymatic oxidation, rolling, and roasting differentially influence oxidative transformation pathways of catechins during tea manufacturing; and (iii) evaluate the relative contributions of raw material characteristics (cultivar type, elevation, and leaf maturity) and processing intensity to the final chemical composition of completed teas.

## 2. Results and Discussion

### 2.1. Chemical Composition at the Green-Tea Processing Stage

As shown in Table 1, a total of nine representative Taiwanese native teas—including Sanxia Bi-Lo-Chung Green Tea (G), Wenshan Paochong Tea (P), High-mountain Oolong Tea (H), Tongding Oolong Tea (D), Muzha Tieh-Kuan-Yin Tea (M), Oriental Beauty Tea (O), Red Oolong Tea (R), Small-leaf Black Tea (SB), and Large-leaf Black Tea (B)—were manufactured independently in triplicate to capture practical manufacturing variability in chemical compositions. All teas were processed by trained tea masters at the Tea and Beverage Research Station (Taoyuan, Taiwan) following the official standards of Taiwanese native teas (CNS 179:2021) [17]. Subsequently, tea samples were collected at four representative processing stages: non-fermented green tea stage (G), fermented stage (F; controlled enzymatic oxidation via withering or stirring), semi-finished tea stage prior to roasting (S), and fully completed tea stage (C) as shown in Table 2.

**Figure 2:**
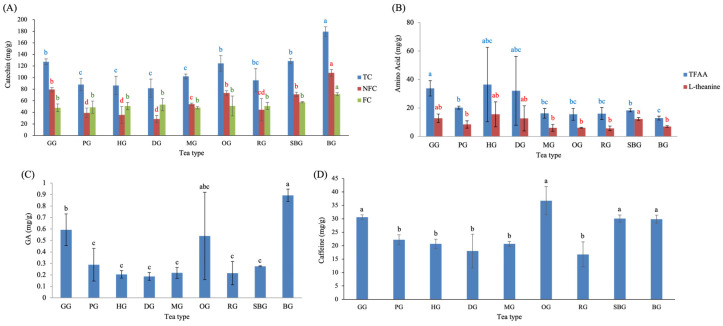
Functional composition of green-tea-processed samples across Taiwanese tea types, including (**A**) catechins, (**B**) amino acids, (**C**) gallic acid, and (**D**) caffeine. Different lowercase letters of the same color indicate significant differences among tea types (*p* < 0.05). Abbreviations: TC, total catechins; NFC, non-free catechins; FC, free catechins; TFAA, total free amino acids; GA, gallic acid.

The green-tea–processed samples (G stage) of the nine Taiwanese tea types exhibited distinct chemical patterns across catechin derivatives, amino acids, GA, and caffeine (Figure 2). Catechins are recognized as the major polyphenolic constituents (flavan-3-ols) of fresh tea leaves [18], and differences in their abundance often reflect cultivar-related characteristics and plucking practices [19]. Consistent with this, Figure 2A showed significant variation in total catechins (TC) among tea types (*p* < 0.05). Teas manufactured from fine-plucked buds—including GG, OG, and SBG—displayed the highest TC levels within the small-leaf types (G, P, H, D, M, O, R, SB). A similar trend was observed for non-free catechins (NFC), in which these three teas also showed significantly higher concentrations within the small-leaf tea group. However, free catechins (FC) exhibited no significant differences among small-leaf tea types. When compared with BG, all catechin parameters—TC, NFC, and FC—were markedly higher in BG than in any small-leaf tea processed at the green-tea stage. This divergence can be partly attributed to cultivar-related differences, as large-leaf cultivars are known to contain higher flavanol/catechin levels than small-leaf cultivars [20], providing a larger substrate pool prior to fermentation.

The contents of total free amino acids (TFAA) and L-theanine in green-tea-processed samples are shown in Figure 2B. GG exhibited the highest TFAA, a result likely reflecting cultivar-specific traits of Chin-Shin-Ganzai, while SBG showed the highest L-theanine content. HG, which included leaf materials collected from 1000, 1400, and 1700 m, displayed progressively higher TFAA and L-theanine levels with increasing altitude, resulting in larger error bars. A similar phenomenon was also observed in DG, where the high-elevation batch (>1200 m) contained substantially higher amino acid levels than batches harvested at 500–600 m. The cooler temperatures, slower leaf growth, and intensified fertilization in high-elevation tea plantations promote amino acid accumulation [21]. Chen et al. [22] suggested that tea grown at high altitude accumulates greater levels of water-extractable compounds—including polyphenols, catechins, and free amino acids—than tea grown at lower elevations. Taken together, these observations are consistent with previously reported altitude-associated trends in amino acid accumulation in tea plants, while the present findings highlight this relationship primarily within the context of the HG and DG samples examined.

Among the small-leaf tea types, GG and OG exhibited the highest GA content (Figure 2C). When BG was included in comparison, BG showed the highest GA content overall, consistent with the trends observed in TC and NFC (Figure 2A). This suggests that plucking tenderness and leaf size likely influence GA biosynthesis and ester hydrolysis. The notably high GA levels in Chin-Shin-Ganzai (GG) and Chin-Shin-Dapan (OG) may also indicate inherent cultivar-specific biochemical characteristics, which warrants future cultivar-based metabolic investigation.

Caffeine levels differed markedly across green-tea-processed samples (Figure 2D). The highest content was detected in OG, GG, SBG, and BG. These trends largely parallel those observed for catechin and GA content, and reflect harvesting maturity, as teas plucked at the bud–leaf stage (e.g., OG harvested as one bud–one leaf) generally retain higher caffeine levels. Lin et al. demonstrated that caffeine content varies systematically with plucking position, with the highest content occurring in the bud and first several leaves and progressively declining in older leaves [23]. These findings indicate that cultivar-related characteristics, leaf maturity, and elevation play a major role in shaping the initial phytochemical composition prior to processing. The substantial differences in catechins, amino acids, GA, and caffeine at the green-tea stage highlight the importance of raw material characteristics as a biochemical foundation that shapes subsequent fermentation and roasting outcomes. It should be noted that variation observed at the green-tea stage also reflects inherent biological variability within tea types, including differences in cultivar, elevation, and leaf maturity among independently manufactured batches. Such variability represents realistic raw-material heterogeneity under practical production conditions and provides the biochemical baseline upon which subsequent processing-driven transformations occur.

### 2.2. Effects of Fermentation on Chemical Composition Across Taiwanese Tea Types

**Figure 3:**
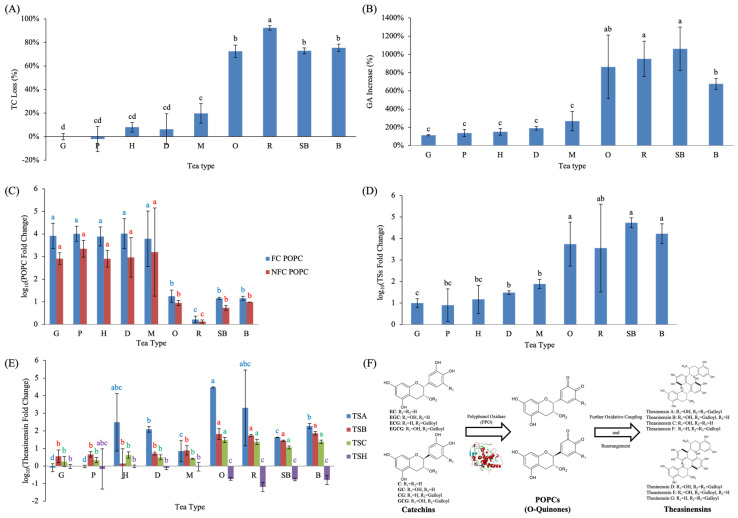
Fermentation-induced changes in major polyphenolic components across nine Taiwanese native teas, including (**A**) TC depletion, (**B**) GA formation, (**C**) POPCs, (**D**) total theasinensins, (**E**) individual theasinensins. Different lowercase letters of the same color indicate significant differences among tea types (*p* < 0.05). (**F**) Schematic illustration of the proposed stepwise oxidative transformation pathway of catechins during tea fermentation. Abbreviations: TC, total catechins; GA, gallic acid; POPCs, paired oxidative polymerization compounds; FC, free catechins; NFC, non-free catechins; TSs, theasinensins; TSA, theasinensin A; TSB, theasinensin B; TSC, theasinensin C; TSH, theasinensin H.

Fermentation induced distinct and tea-type specific changes in catechins, GA, amino acids, and downstream oxidative polymerization products. When interpreting fermentation-induced changes, it should be emphasized that differences among tea types reflect both processing intensity and inherent biological variability in raw materials, including cultivar, elevation, and leaf maturity. To evaluate the extent of catechin oxidation during processing, the ratio of F to green-tea-processed (G) samples was calculated for each tea type (Figure 3A). Five lightly fermented teas—including G, P, H, D, and M—showed minimal TC loss (≤20%), indicating that withering or mild enzymatic oxidation caused only limited degradation of monomeric catechins. In contrast, heavily fermented teas exhibited pronounced catechin depletion: O, SB, and B showed ~70% TC loss, whereas R displayed the highest reduction (~92%). These findings align with previous research showing that approximately 75% of catechins in tea leaves undergo enzymatic oxidation and polymerization during fermentation, forming secondary polyphenolic compounds such as theaflavins, thearubigins, theasinensins, theacitrins, and oolongtheanins, which contribute to the characteristic color and flavor of fermented teas [8]. Moreover, studies have demonstrated that teas with a higher initial content of esterified catechins (particularly EGCG and ECG) generate greater levels of theaflavins during fermentation, resulting in deeper infusion color and improved sensory quality [20]. Previous studies have shown that during fermentation, the oxidation of tea polyphenols proceeds in a predictable way, with the reaction rate governed by molecular structure and oxygen concentration [15]. These results imply that fermentation intensity is the primary driver of catechin oxidation and polymerization.

GA increased markedly following fermentation in all tea types, with the largest increases observed in SB, followed by R and O (Figure 3B). Previous studies have demonstrated that EGCG and ECG readily undergo hydrolysis and oxidative degradation, yielding GA and de-galloylated catechins under oxygen-rich conditions [24,25]. The accumulation of GA thus reflects both the degree of catechin biotransformation and the progression of fermentation. Furthermore, GA contributes to the bitterness, astringency, and antioxidant capacity of fermented teas [26,27], and is increasingly recognized as a potential biochemical indicator of fermentation intensity and tea quality [28]. Overall, these findings suggest that GA formation is not only a degradation byproduct, but also an integral component of the oxidative transformation pathway, playing a significant role in shaping both the chemical functionality and sensory characteristics of fermented teas.

LC–MS/MS analysis revealed clear differences in the relative abundance of paired oxidative polymerization compounds (POPCs), evaluated in a semi-quantitative manner, among tea types (Figure 3C). Both FC and NFC POPCs showed similar patterns. Lightly fermented teas (G, P, H, D, M) displayed the largest relative increases, whereas heavily fermented teas (O, SB, B) accumulated only about one-quarter as much, and R showed the lowest levels overall. Previous studies indicate that catechins are oxidized to quinone intermediates, which subsequently couple and polymerize into higher-order pigments such as theaflavins, thearubigins, and theabrownins [8,16]. These oxidative polymerization products significantly contribute to tea’s color and taste [29]. This stepwise progression supports the interpretation that POPCs represent early stage oxidative coupling products which, under stronger fermentation conditions, tend to decrease as downstream oxidation products such as theasinensins and theaflavin/thearubigin-type pigments accumulate.

Theasinensins (TSs), another major group of oxidation-derived catechin dimers, were substantially affected by fermentation, as shown in Figure 3D,E. The teas subjected to stronger fermentation, including O, R, SB and B, exhibited the largest relative increases in total TSs. This pattern indicates that prolonged enzymatic oxidation favors more extensive coupling between catechin quinone intermediates. The individual TS compounds, however, did not respond uniformly across tea types. Theasinensin A (TSA) increased significantly in all teas except G and P, and the magnitude of increase was greatest in O. Both theasinensin B (TSB) and theasinensin C (TSC) also increased across all tea types, with particularly pronounced increases in O, R, SB and B. In contrast, theasinensin H (TSH), an isomer of TSB, showed marked decreases in these heavily fermented teas, suggesting that TSH may be involved in further oxidative transformations during later stages of fermentation.

The identification of catechin-derived oxidation products in this study is consistent with previously reported MS/MS fragmentation behaviors. Characteristic fragment ions associated with neutral losses of galloyl moieties, together with fragmentation patterns indicative of retro-Diels–Alder cleavage of the flavan-3-ol backbone, have been widely reported as diagnostic features for catechin-derived oxidative dimers, including early stage coupling products and theasinensins, in fermented teas. In targeted LC–MS/MS workflows, these established fragmentation features with retention time matching provides reliable confirmation of compound identity. The application of such validated MS/MS criteria supports the assignment of oxidative catechin dimers detected across different fermentation stages in the present study [8,15,16].

Taken together, the coordinated relative changes in catechins, POPCs, and theasinensins support a stepwise oxidative transformation pathway during tea fermentation. Catechins serve as the primary substrates for enzymatic oxidation, generating quinone intermediates that undergo early oxidative coupling to form paired oxidative polymerization compounds (POPCs). Under stronger or prolonged fermentation conditions, these early stage coupling products are further transformed into more structurally defined and stable catechin dimers, such as theasinensins. This proposed relationship and progression are schematically illustrated in Figure 3F, which integrates representative coupling motifs and oxidation pathways reported in the literature [8,16].

Compared with previous studies that primarily focused on single tea categories or final commercial products, the present study extends current knowledge by providing a process-resolved comparison of multiple Taiwanese native teas across defined manufacturing stages. Earlier reports have extensively characterized catechin oxidation products, such as theaflavins and theasinensins, mainly in black or oolong teas after fermentation [8,15,16]. In contrast, the staged sampling strategy employed here enables the direct observation of early oxidative coupling events, reflected as paired oxidative polymerization compounds (POPCs), prior to their further transformation into more structurally defined pigments under stronger fermentation conditions. Moreover, while previous studies have reported fermentation-dependent trends in catechin depletion and gallic acid formation, these relationships were often inferred from comparisons among finished teas produced under different conditions [15,30]. By tracking identical tea types through sequential processing steps, the present study demonstrates that POPCs accumulate preferentially under mild fermentation and decline as fermentation intensifies, thereby refining the temporal framework of catechin oxidation pathways during tea manufacturing. Together, these findings do not contradict previous reports but rather nuance them by integrating early stage intermediates and processing dynamics into a unified transformation model, providing a more mechanistic understanding of how fermentation intensity and processing structure jointly shape the chemical profiles of Taiwanese native teas.

**Figure 4:**
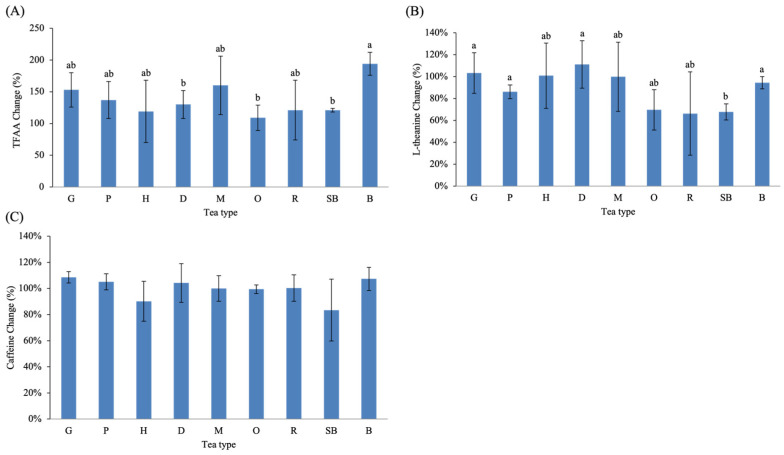
Fermentation-induced changes in nitrogenous compounds and caffeine across Taiwanese native teas, including percentage changes in (**A**) TFAA, (**B**) L-theanine, and (**C**) caffeine. Different lowercase letters indicate significant differences among tea types (*p* < 0.05). Abbreviations: TFAA, total free amino acids.

Fermentation also influenced nitrogenous compounds, particularly free amino acids (Figure 4A,B). TFAA increased in all tea types relative to their green-tea-stage counterparts. Among them, B exhibited the highest mean increase (+194%), followed by M (+160%) and G (+153%). Although numerical values varied across tea types, no statistically significant differences were detected among the small-leaf teas, indicating that the observed TFAA increases within these teas were comparable in magnitude.

In contrast, L-theanine displayed both increases and decreases depending on tea type. Notably, O, R, and SB showed the largest decreases in L-theanine content after fermentation. Although these differences were not statistically significant across all comparisons, these three teas share processing characteristics involving prolonged withering. For example, O undergoes ~3 h of solar withering in warm early summer conditions followed by extended indoor withering and repeated stirring, resulting in substantial moisture loss before pan-firing. R also involves the longest total withering time among partially fermented teas, combining solar withering and extended indoor withering/stirring cycles. SB is subjected to overnight withering immediately after harvest and additional rolling and stirring steps prior to fixation. This is consistent with previous reports indicating that withering, rolling, and fermentation promote the hydrolysis of tea-leaf proteins into free amino acids. However, these amino acids often decline during fermentation, likely due to their further conversion into downstream metabolites.

Caffeine remained relatively stable during fermentation (Figure 4C). Across all teas, changes did not exceed 20%, and no association with fermentation intensity was observed. This result is consistent with previous findings showing that caffeine is one of the most stable constituents in tea and is largely unaffected by fermentation processes [31,32]. Variations in caffeine content are more strongly influenced by environmental factors—such as growing region, climate, and harvest time—rather than by manufacturing steps [33]. These results therefore counter the common misconception that higher fermentation reduces caffeine levels.

### 2.3. Effects of Roasting on Chemical Composition

**Figure 5:**
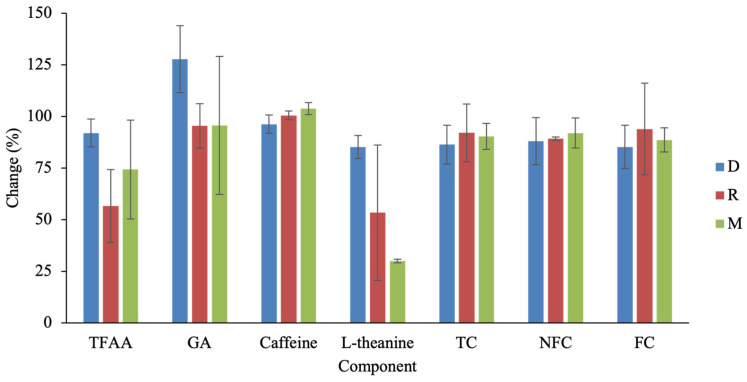
Percentage changes in major components from S to C in three Taiwanese tea types. Abbreviations: TFAA, total free amino acids; GA, gallic acid; TC, total catechins; NFC, non-free catechins; FC, free catechins.

Roasting further modified the chemical composition during the transition from the semi-finished (S) to completed (C) processing stage (Figure 5). It should be noted that only D, M, and R were included in the S–C comparison because these tea types involve a clearly defined and independent roasting step in their manufacturing protocols, allowing a distinct S to be sampled prior to roasting. Although not all changes reached statistical significance, each tea type exhibited a distinct response pattern; therefore, the results are discussed here in terms of relative trends rather than statistically significant differences. In D, roasting was associated with a marked increase in GA, whereas most other components showed moderate decreases. Across all three teas, TFAA and L-theanine exhibited the greatest reductions after roasting, a trend consistent with the known susceptibility of nitrogenous compounds to thermal degradation, deamination, or Maillard reactions during pro-longed heating.

Roasting conditions differed substantially among tea types and likely contributed to their compositional outcomes. D undergoes an average of 18.3 h of roasting, reaching temperatures up to 115 °C. M experiences the most intensive treatment—over 35 h of cumulative roasting with peak temperatures around 120 °C—consistent with its pronounced losses of TFAA and L-theanine and its GA formation. R, in contrast, has the shortest roasting duration (~16 h, max 105 °C); however, its TFAA reduction exceeded that of D. This discrepancy may be explained by differences in process structure: D is roasted in 9–10 short cycles spread across several days, allowing intermittent moisture redistribution and surface cooling, whereas R undergoes only two long roasting cycles (~8 h each). The limited cooling intervals in R reduce opportunities for moisture equilibration and may intensify thermal stress on nitrogenous compounds, resulting in greater amino acid loss despite milder temperature conditions.

### 2.4. Chemical Composition of Completed Teas (C) and the Influence of Rolling

**Figure 6:**
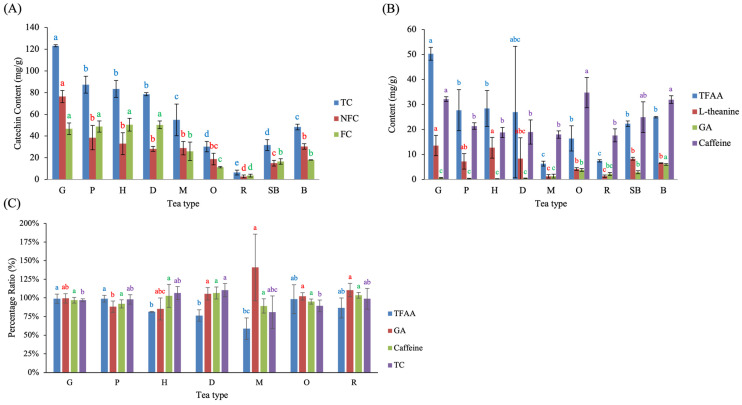
Chemical composition of completed Taiwanese native teas and changes introduced by post-fermentation processing. Panels show (**A**) catechin profiles, (**B**) contents of TFAA, L-theanine, GA, and caffeine, and (**C**) percentage ratios of major components after pan-firing and rolling. Different lowercase letters of the same color indicate significant differences among tea types (*p* < 0.05). Abbreviations: TC, total catechins; NFC, non-free catechins; FC, free catechins; TFAA, total free amino acids; GA, gallic acid.

The chemical composition of C varied markedly across Taiwanese tea types (Figure 6). Among all samples, G exhibited the highest levels of TC, NFC, FC, TFAA, L-theanine, and caffeine (Figure 6A,B). The distribution patterns of individual catechins observed in the present study are generally consistent with those reported in previous studies using solvent-based extraction methods [15,30]. For example, green teas reported by Zuo et al. [30] showed a predominance of non-free catechins (NFC), whereas oolong and black teas exhibited lower total catechin contents, reflecting progressive oxidative transformation during fermentation. Although absolute catechin concentrations are not directly comparable due to differences in extraction solvents and data normalization, the relative dominance of NFC in green teas and their gradual depletion with increasing fermentation degree align well with the trends observed in the present hot-water-extracted samples.

In contrast, GA was most abundant in B (6.03 mg/g), followed by O (3.84 mg/g), SB (2.92 mg/g), and R (2.21 mg/g), as shown in Figure 6B. Notably, the GA contents observed in the present study are comparable to values reported for corresponding tea categories in previous studies [30]. For example, the GA content of Sanxia Bi-Lo-Chung green tea (G, 0.66 mg/g) was similar to that reported for Meifoo green tea (0.74 mg/g). Likewise, the GA level in Muzha Tieh-Kuan-Yin tea (M, 1.35 mg/g) closely matched that reported for Fujian oolong tea (1.42 mg/g), and the GA content of Small-leaf Black Tea (SB, 2.92 mg/g) was comparable to that reported for Fujian black tea (2.06 mg/g). Despite differences in extraction conditions and data expression, these comparisons are consistent with an increasing trend in GA content from non-fermented green teas to semi-fermented oolong teas and fully fermented black teas. This agreement supports the analytical reliability of the present measurements and highlights fermentation degree as a key determinant of GA accumulation. Minor variation observed among batches within the same tea type likely reflects differences in leaf maturity and raw material selection, which are inherent to artisanal tea production rather than analytical uncertainty. In addition, one batch of D showed larger variation in Figure 6B, likely due to the use of more mature leaf material at harvest. Caffeine content was highest in G, O, and B—three teas produced from bud-rich raw materials—indicating that plucking maturity, rather than manufacturing steps, is the dominant determinant of caffeine levels. Consistent with previous observations, Zuo et al. [30] also suggested that caffeine content does not scale proportionally with fermentation degree, implying that factors other than oxidation intensity play a major role in determining caffeine levels in tea.

To evaluate how rolling affects tea chemistry, the ratio of C to its previous processing stage was calculated (Figure 6C). For G, P, H, and O, the comparison was made between C and the fermentation stage (F). For D, M, and R, the comparison was made between C and the S. Rolling and ball-rolling produced heterogeneous effects on catechins. G, P, and R showed slight decreases in TC (≤5%), whereas H and D exhibited modest increases. M and O showed the largest TC reductions (≥10%), suggesting that post-pan-firing holding in O, as well as the traditional cloth-ball rolling applied in M, may have promoted additional catechin oxidation. For TFAA, G, P, and O exhibited minimal changes (<3%), whereas H, D, and R showed larger reductions of 19%, 24%, and 14%, respectively. M experienced the most substantial decline (40%), indicating that extended ball-rolling and heat accumulation may contribute to accelerated amino acid loss. GA increased most prominently in M (+41%), consistent with the observed loss of TC in this tea. Caffeine showed only minor fluctuations (~10%) across all tea types, with no significant differences among them, reaffirming its relative stability during rolling.

Taken together, the results demonstrate that catechins are sensitive components to rolling-related thermal and mechanical stress, particularly in teas processed with extended holding or traditional cloth-ball rolling. Amino acids also show measurable reductions under these conditions, whereas caffeine remains largely unaffected.

## 3. Materials and Methods

### 3.1. Chemicals and Reagents

(−)-Epigallocatechin (EGC, ≥95%), (−)-Epicatechin (EC, ≥98%), (−)-Epigallocatechin gallate (EGCG, ≥95%), (−)-Catechin (C, ≥98%), (−)-Gallocatechin gallate (GCG, ≥98%), (−)-Epicatechin gallate (ECG, ≥98%), (−)-Gallocatechin (GC, ≥98%), (−)-Catechin gallate (CG, ≥98%), GA, caffeine, L-theanine, and polyvinylpolypyrrolidone (PVPP) were all purchased from Sigma-Aldrich (St. Louis, MO, USA). Stannous chloride (SnCl_2_) was obtained from Alfa Aesar (Ward Hill, MA, USA). Ninhydrin, acetonitrile (LC grade), formic acid (LC grade, 85%), potassium sodium tartrate tetrahydrate, ferrous sulfate heptahydrate (FeSO_4_·7H_2_O), disodium hydrogen phosphate dodecahydrate, and potassium dihydrogen phosphate were purchased from Merck (Darmstadt, Germany). Borate, o-phthalaldehyde (OPA), and 9-fluorenylmethyl chloroformate (FMOC) were obtained from Agilent Technologies (Santa Clara, CA, USA).

### 3.2. Tea Processing and Sample Collection

Nine representative Taiwanese native teas—G, P, H, D, M, O, R, SB, and B—were manufactured at the Tea and Beverage Research Station, Ministry of Agriculture (Taoyuan, Taiwan). For each tea type, independent production batches were manufactured in triplicate using cultivars officially recognized for that category. Consequently, variation among batches reflects the natural differences in cultivar, leaf maturity, and elevation commonly encountered within each tea type under practical manufacturing conditions. These sources of variability were intentionally retained to reflect authentic manufacturing conditions, and data interpretation therefore focused on processing stage-dependent trends at the tea-type level rather than on cultivar-, elevation-, or leaf-maturity-specific effects. All manufacturing procedures followed the official standards of Taiwanese native teas (CNS 179:2021) [17].

Sampling was conducted at representative stages of tea processing to reflect distinct manufacturing steps. The processing parameters applied in this study, including withering duration and roasting conditions, were selected to reflect standard commercial practices for the respective Taiwanese tea types, rather than to optimize specific chemical outcomes.

1. Green tea stage (non-fermented group, G) was freshly plucked tender shoots and pan-fired immediately after harvest to inactivate endogenous enzymes and prevent enzymatic browning. The leaves were not rolled but directly dried to preserve their original green characteristics.
2. Fermentation stage (fermented group, F), corresponding to the enzymatic fermentation (oxidative fermentation) stage defined in the Abstract, consisted of withering or stirring of fresh tea leaves to induce enzymatic oxidation (tea fermentation). Withering reduced moisture and facilitated the formation of aroma precursors, whereas stirring redistributed leaf moisture and caused partial cell rupture, thereby enhancing polyphenol oxidase–catechin reactions and regulating the degree of fermentation. After fermentation was achieved, the leaves were pan-fired without rolling to inactivate enzymes.
3. Semi-finished tea stage (S) represented the intermediate samples of teas that included a roasting process, namely D, M, and R. These samples were collected after shaping (rolling) but before roasting to evaluate the influence of roasting on the chemical composition.
4. Completed tea stage (C) consisted of tea samples that had completed the full manufacturing process for each tea type.

### 3.3. Tea Extraction

Ground tea powder (0.5 g) was extracted with 45 mL of deionized water at 90 °C for 20 min, filtered, and adjusted to 50 mL. Tea samples (3 g) were dried at 105 °C in an oven to constant weight. Moisture content was calculated as weight loss (%).

### 3.4. HPLC Analysis of Catechins and Caffeine

Quantification of individual catechins and caffeine followed the Chinese National Standard CNS 15022 (N6384) [34]. The method of Zuo et al. for determining catechins, caffeine, and GA in tea was used with minor chromatographic modifications [30].

Analyses were performed using an Agilent 1260 HPLC system (Agilent Technologies, Santa Clara, CA, USA) equipped with a PUROSPHER STAR RP-18(E) column (250 × 4 mm, 5 µm; Merck KGaA, Darmstadt, Germany) and a diode-array detector (Agilent Technologies, Santa Clara, CA, USA) set at 280 nm. The flow rate was maintained at 1.0 mL min^−1^ with an injection volume of 10 µL. Solution A (0.1% formic acid in water) and solution B (acetonitrile) were used as mobile phases. The elution gradient was programmed as follows: 0–5 min, linear increase from 0 to 10% B; 5–15 min, isocratic at 10% B; 15–29 min, linear increase to 20% B; 29–35 min, to 22% B; and 35–40 min, to 25% B. Calibration curves for catechins, gallic acid, and caffeine were constructed using analytical standards over appropriate concentration ranges, with coefficients of determination (R^2^) greater than 0.99. All samples were analyzed in triplicate.

### 3.5. HPLC Analysis of L-Theanine and TFAA

Analyses were performed using an Agilent 1260 HPLC system equipped with a Zorbax Eclipse-AAA column (150 × 4.6 mm, 5 µm; Agilent Technologies, Santa Clara, CA, USA) and a fluorescence detector (Agilent Technologies, Santa Clara, CA, USA) set at 338 nm. The flow rate was maintained at 2.0 mL min^−1^ with an injection volume of 10 µL. Solution A (10 mM ammonium ethanoate) and solution B (methanol:acetonitrile:water = 45:45:10, *v*/*v*/*v*) were used as mobile phases. The separation method followed Zheng et al. [35] with minor modifications. The contents of L-theanine and TFAA were quantified using external calibration curves established with analytical standards over appropriate concentration ranges, with coefficients of determination (R^2^) greater than 0.99. Total free amino acids were determined by HPLC-fluorescence analysis of derivatized free amino acids and are presented as total free amino acid content rather than individual amino acid composition. Results were expressed on a dry weight basis.

### 3.6. LC–MS/MS Analysis

LC–MS/MS analyses were conducted in a targeted mode by an external analytical facility using validated protocols, with chromatographic separation performed on an Agilent LC system (Agilent Technologies, Santa Clara, CA, USA). Chromatographic separation was performed using a Poroshell 120 EC-C18 column (2.1 × 100 mm, 2.7 µm; Agilent Technologies, Santa Clara, CA, USA). The flow rate was maintained at 0.4 mL min^−1^ with an injection volume of 10 µL. Solution A (0.1% formic acid in water) and solution B (methanol) were used as mobile phases. The elution gradient was programmed as follows: 0–2 min, 5–20% B; 2–8 min, isocratic at 20% B; 8–12 min, linear increase to 60% B; 12–15 min, isocratic at 60% B; 15–19 min, linear increase to 95% B; 19–24 min, isocratic at 95% B; and 24–27 min, return to 5% B and equilibrate to 30 min. Mass spectrometric detection was carried out using a triple quadrupole mass spectrometer (Agilent Technologies, Santa Clara, CA, USA) equipped with an electrospray ionization (ESI) source (Agilent Technologies, Santa Clara, CA, USA). Compound identification was based on predefined MS/MS transitions and retention time matching with reference standards or literature-reported fragmentation patterns [8,15,16]. LC–MS/MS analysis was specifically applied for the identification and quantification of theasinensins and operationally defined paired oxidative polymerization compounds (POPCs), representing early stage catechin-derived oxidative coupling products. Theasinensins were quantified using external calibration curves constructed over a concentration range of 0.01–100 μg/mL, with coefficients of determination (R^2^) greater than 0.99, following validated protocols provided by the external analytical facility. As paired oxidative polymerization compounds (POPCs) represent an operational grouping of early catechin-derived oxidative coupling products for which authentic reference standards are not universally available, POPCs were evaluated in a semi-quantitative manner based on LC–MS/MS response. Accordingly, POPC levels are reported as relative changes (fold change) across processing stages rather than as absolute concentrations. The analytical outputs consisted of compound-specific identification and quantitative results, which were used for compound confirmation and data interpretation.

### 3.7. Statistical Analysis

All measurements were conducted on three independently manufactured batches per tea type (*n* = 3) and expressed as mean ± standard deviation. These independent batches represent practical manufacturing variability within each tea type, rather than strict biological replicates derived from a uniform biological source. Statistical analyses were performed using CoStat version 6.400 (CoHort Software, Monterey, CA, USA). Differences among tea types at each processing stage were evaluated using one-way analysis of variance (ANOVA), followed by Duncan’s multiple range test, which is commonly applied in food science studies for post hoc comparisons when sample sizes are limited, to determine significant differences among means at *p* < 0.05. Accordingly, statistical analyses were used to characterize process-related differences among tea types across manufacturing stages, with results interpreted in the context of manufacturing conditions and raw materials variability rather than strictly controlled biological variance.

## 4. Conclusions

This study provides a comprehensive, stage-based comparison of nine representative Taiwanese native teas, revealing how cultivar traits and processing steps collectively shape their chemical composition. At the green-tea stage, large-leaf cultivars contained higher catechin and gallic acid (GA) levels than small-leaf cultivars, while bud-rich and high-elevation materials showed elevated free amino acids and L-theanine. Fermentation induced tea-type-specific transformations: lightly fermented teas exhibited limited catechin loss, whereas heavily fermented teas showed extensive catechin depletion accompanied by substantial GA formation and marked increases in theasinensins. The accumulation of paired oxidative polymerization compounds (POPCs), as defined in this study, was greatest in lightly fermented teas, reflecting early stage catechin oxidation, whereas heavily fermented teas likely converted these intermediates into higher-order oxidation products. Free amino acids generally increased during fermentation, whereas L-theanine decreased markedly in teas likely related to prolonged withering. Caffeine remained highly stable across all processing stages. Roasting of semi-finished teas further reduced amino acids and L-theanine while increasing GA depending on roasting structure and temperature. In completed teas, Sanxia Bi-Lo-Chung Green Tea exhibited the highest catechin and amino acid levels, whereas Large-leaf Black Tea had the highest GA content. Overall, catechins and amino acids were the most sensitive to oxidative, mechanical, and thermal processing, while caffeine remained largely unaffected. These findings establish a chemical framework that links cultivar selection and processing decisions to final tea quality, offering valuable guidance for optimizing Taiwanese native tea production and enhancing functional phytochemical profiles. From an applied perspective, this stage-resolved compositional framework may assist tea manufacturers in tailoring processing strategies to achieve the desired chemical profiles and may support product differentiation and quality communication in industry and retail settings. It should be noted that cultivar, growing environment, and processing intensity were not independently controlled in this study, and future work employing controlled experimental designs and targeted compound identification would further refine mechanistic understanding.

## Figures and Tables

**Table 1:** Taiwanese native tea types and corresponding cultivars.

Sample ID	Tea Type	Cultivar
G1	Sanxia Bi-Lo-Chung Green Tea	Chin-Shin-Ganzai
G2	Sanxia Bi-Lo-Chung Green Tea	Chin-Shin-Ganzai
G3	Sanxia Bi-Lo-Chung Green Tea	Chin-Shin-Ganzai
P1	Wenshan Paochong Tea	TTES No. 12 (Jhinshuan)
P2	Wenshan Paochong Tea	Chin-Shin-Oolong
P3	Wenshan Paochong Tea	TTES No. 20
H1	High-mountain Oolong Tea	TTES No. 12 (Jhinshuan)
H2	High-mountain Oolong Tea	Chin-Shin-Oolong
H3	High-mountain Oolong Tea	Chin-Shin-Oolong
D1	Tongding Oolong Tea	Chin-Shin-Oolong
D2	Tongding Oolong Tea	Chin-Shin-Oolong
D3	Tongding Oolong Tea	TTES No. 12 (Jhinshuan)
M1	Muzha Tieh-Kuan-Yin Tea	Tieh-Kuan-Yin
M2	Muzha Tieh-Kuan-Yin Tea	Tieh-Kuan-Yin
M3	Muzha Tieh-Kuan-Yin Tea	Tieh-Kuan-Yin
O1	Oriental Beauty Tea	Chin-Shin-Dapan
O2	Oriental Beauty Tea	Chin-Shin-Dapan
O3	Oriental Beauty Tea	Chin-Shin-Dapan
R1	Red Oolong Tea	TTES No. 12 (Jhinshuan)
R2	Red Oolong Tea	TTES No. 20
R3	Red Oolong Tea	FoShou
B1	Large-leaf Black Tea	TTES No. 18 (Ruby)
B2	Large-leaf Black Tea	TTES No. 18 (Ruby)
B3	Large-leaf Black Tea	TTES No. 18 (Ruby)
SB1	Small-leaf Black Tea	Chin-Shin-Oolong
SB2	Small-leaf Black Tea	Chin-Shin-Oolong
SB3	Small-leaf Black Tea	Chin-Shin-Oolong

**Table 2:** Processing groups and assigned sample codes for each tea type.

Sample ID	Sanxia Bi-Lo-Chung Green Tea (G)	Wenshan Paochong Tea (P)	High-Mountain Oolong Tea (H)	Tongding Oolong Tea (D)	Muzha Tieh-Kuan-Yin Tea (M)	Oriental Beauty Tea (O)	Red Oolong Tea (R)	Small-Leaf Black Tea (SB)	Large-Leaf Black Tea (B)
Green tea stage (G)	GG	PG	HG	DG	MG	OG	RG	SBG	BG
Fermentation stage (F)	GF	PF	HF	DF	MF	OF	RF	-	-
Semi-finished tea stage (S)	-	-	-	DS	MS	-	RS	-	-
Completed tea stage (C)	GC	PC	HC	DC	MC	OC	RC	SBC	BC

## Data Availability

Data are contained within the article.
